# ‘MALADAPTIVE DAYDREAMING’: An introduction to a new condition

**DOI:** 10.1192/j.eurpsy.2022.473

**Published:** 2022-09-01

**Authors:** C. Thorburn

**Affiliations:** Peterborough City Hospital, The Cavell Centre, Peterborough, United Kingdom

**Keywords:** DSM-5, mental health, maladaptive daydreaming, ICD-10

## Abstract

**Introduction:**

Daydreaming is a normal, very common experience in childhood and adulthood. However, a new phenomenon – termed ‘Maladaptive Daydreaming (MD)’ – which takes daydreaming to an extreme form, is currently being investigated. Maladaptive Daydreaming is not listed as an official disorder in the ICD-10 or DSM-5 presently.

**Objectives:**

To review current literature on MD and explore whether MD could be acknowledged and classified as a real psychiatric disorder.

**Methods:**

Data gathered via academic papers found through reliable sites, such as, Ovid, PubMed and Cochrane; through articles, videos and online forums to gather patients’ perspectives.

**Results:**

There is enough information and literature available to create specific criteria to qualify a diagnosis of MD in patients. Possible aetiologies of Maladaptive Daydreaming have been identified. There has also been exploration into treatment options.

**Conclusions:**

There is sufficient evidence for Maladaptive Daydreaming to be classified as an official disorder. Being included in the ICD-10 and DSM-5 would motivate research, expand identification of this disorder in patients, and advance access to help and support for patients.

**Disclosure:**

No significant relationships.

